# Touching the social robot PARO reduces pain perception and salivary oxytocin levels

**DOI:** 10.1038/s41598-020-66982-y

**Published:** 2020-06-17

**Authors:** Nirit Geva, Florina Uzefovsky, Shelly Levy-Tzedek

**Affiliations:** 1 Recanati School for Community Health Professions, Department of Physical Therapy, Faculty of Health Sciences, Ben-Gurion University of the Negev, Beer-Sheva, Israel; 2 Zlotowski Center for Neuroscience, Ben-Gurion University of the Negev, Beer-Sheva, Israel; 3 Department of Psychology, Ben-Gurion University of the Negev, Beer-Sheva, Israel; 4 Freiburg Institute for Advanced Studies (FRIAS), University of Freiburg, Freiburg, Germany

**Keywords:** Emotion, Social behaviour, Empathy, Pain, Stress and resilience

## Abstract

Human-human social touch improves mood and alleviates pain. No studies have so far tested the effect of human-robot emotional touch on experimentally induced pain ratings, on mood and on oxytocin levels in healthy young adults. Here, we assessed the effect of touching the robot PARO on pain perception, on mood and on salivary oxytocin levels, in 83 young adults. We measured their perceived pain, happiness state, and salivary oxytocin. For the 63 participants in the PARO group, pain was assessed in three conditions: Baseline, Touch (touching PARO) and No-Touch (PARO present). The control group (20 participants) underwent the same measurements without ever encountering PARO. There was a decrease in pain ratings and in oxytocin levels and an increase in happiness ratings compared to baseline only in the PARO group. The Touch condition yielded a larger decrease in pain ratings compared to No-Touch. These effects correlated with the participants’ positive perceptions of the interaction with PARO. Participants with higher perceived ability to communicate with PARO experienced a greater hypoalgesic effect when touching PARO. We show that human-robot social touch is effective in reducing pain ratings, improving mood and - surprisingly - reducing salivary oxytocin levels in adults.

## Introduction

Social interaction is one of the most basic survival needs of humans^1^. Both in childhood^2–5^ and in older ages^6–8^, the impact of social connections on health seems to be crucial. For example, poor social relationships and social isolation were associated with high incidence of general morbidity^9,10^, stress disorders^11,12^ and chronic pain^11,13,14^. Close relationships, however, were found to be a protective factor against stress and pain disorders^15–18^. Close interpersonal relationships often involve emotional touch, which may act as a mediating factor in the effect of social relationships on pain relief. Emotional touch is defined as a pleasant touch between two humans^19^. Emotional touch may include active touching (i.e. stroking another person), passive touching (being touched by another person) or dyadic touching (i.e. hand holding)^20,21^. Indeed, several studies have found that handholding^22^ and hugging^23^ reduce the physiological and psychological response to stress among men and women. It was suggested that empathic abilities of both the person touching and the person touched play a fundamental role in this effect^24^. Emotional touch also stimulates the hypothalamic-pituitary system to secrete oxytocin^20,25^, a hormone that has been characterized as having a central role in mediating feelings of love, social attachment and communication in both animals and humans^26–29^. In an animal study, there was an increase in pain thresholds following petting, as well as following injection of oxytocin. In both situations (petting or injection of oxytocin), the effect on the pain threshold disappeared with the administration of an oxytocin antagonist^30^. Similarly, in humans, Kreuder *et al*.^31^, recently demonstrated that administration of nasal oxytocin enhances the pain-relieving effects of social support in romantic couples. In addition, it was found that being touched by another person^32^ and handholding with a spouse^33,34^ induce a reduction in pain ratings among women. The level of the analgesic effect when holding a partner’s hand was associated with the toucher’s empathic tendencies^33^. These studies suggest that emotional touch may lead to decreased sensitivity to pain that may be associated with the release of oxytocin. How, then, can the beneficial effect of emotional touch on the perception of pain be provided to individuals who do not have access to it? One way to fill this need may be through a social robot. A social robot may take on a human-like^35,36^ or a pet-like appearance, or move like one e.g^37,38^.. It is designed to create social relationships with people^39^ for either entertainment^40^, education^41^, or for therapeutic purposes^42,43^. Shibata^44^ developed a seal-like robot named PARO designed to elicit a feeling of social connection. Interaction with PARO was found to improve mood^45,46^, and to reduce stress and anxiety of older people, and of individuals with dementia^45–47^, as well as to improve the mood of pediatric patients^48^. In one study, participants interacted with PARO for the duration of one year, during which the effects on mood were maintained^46^. In addition, interaction with PARO^49^ and with a humanoid robot^50^ was found to reduce stress, anxiety and pain levels during medical procedures (chemotherapy among women and vaccination among children, respectively). However, a recent review concluded that better methodology and measures are needed to draw conclusions about the effect of human-robot social interactions on pain^51^. Indeed, no controlled studies specifically examined the effect of the robot’s *touch* as opposed to the robot’s *presence*, without any physical contact, on the perception of pain. In addition, no controlled studies have examined the effect of human-robot social interaction on either oxytocin secretion or on experimentally induced pain ratings.

The aim of the current experiment was, therefore, to examine the effect of interaction with the social robot PARO on pain perception, emotional state, and salivary oxytocin levels. Specifically, we examined, in a group of men and women: (1) What is the effect of human-robot interaction on the (a) happiness state, (b) salivary oxytocin levels and (c) pain perception? (2) What is the effect of social robot’s *touch* vs. the social robot’s *presence* on pain perception? (3) Are there correlations between pain perception and (a) the level of salivary oxytocin; and (b) the participant’s perception of the interaction with the robot?

## Methods

### Participants

Eighty-three healthy adults (42 female, 41 male; age: 25.1 ± 2.7 years old (mean ± STD)) were allocated using a computer-generated simple random sampling, into one of two groups: the PARO-Interaction (PARO) group (63 participants, 32 female, 31 male; 25.2 ± 2.4 years old), or the control group (20 participants, 10 female, 10 male; 24.4 ± 2.2 years old). The participants were recruited by advertisements posted throughout the university campus and on social media. Exclusion criteria were acute or chronic pain, present or previous pathology in the arms (testing site), bruises or any other skin lesions on the arms, diseases causing potential neural damage (e.g., diabetes), systemic and mental illnesses (e.g., anxiety disorders, major depression, bipolar disorder), and communication disabilities. Written informed consent was obtained from all the participants. Written informed consent for publication of identifying images in an online open-access publication was obtained from the persons photographed. The experiment was approved by the institutional review board of Ben-Gurion University. All experimental procedures were performed in accordance with this ethical approval.

### Study design

The 63 participants enrolled in the PARO group comprised the main study group. These participants interacted with PARO and were tested before, during and after the interaction in a within-subjects design. In order to rule out any carry-over effects – that is, the effects of repeated pain measurements on participants’ pain perception – we included a control group of 20 participants, who did not interact with PARO. The control group’s size was informed by;^52–54^ the PARO group was larger, to account for the randomized allocation into different experimental sequences within it (see details below). Differences between participants in the PARO and the control groups were calculated as a between-subject analysis.

### Equipment

#### PARO robot

PARO is a therapeutic robot baby harp seal, manufactured by the Intelligent System Research Institute of Japan’s National Institute of Advanced Industrial Science and Technology. PARO is intended to have a calming effect and to elicit emotional responses in patients^55^. It is outfitted with dual 32-bit processors, three microphones, twelve tactile sensors covering its fur, touch-sensitive whiskers, and a system of motors and actuators that move its limbs and body. The robot responds to petting by moving its tail and opening and closing its eyes. It seeks out eye contact and produces sounds similar to a real baby seal^55^. PARO was classified as a Class 2 medical device by U.S. regulators in 2009, and is completely safe for human interaction^56^.

#### Thermal stimulator

Heat stimuli were delivered using a Peltier-based computerized thermal stimulator (TSA II, Medoc Ltd., Ramat-Ishai, Israel), with a 3 × 3 cm contact probe that was attached to the ventral aspect of the non-dominant forearm by means of a Velcro band. The baseline temperature of the stimulator was set to 35 °C for all the tests. The stimulator is accurate to within ±0.3 °C.

#### Visual analog scale (VAS)

**Figure 1:**
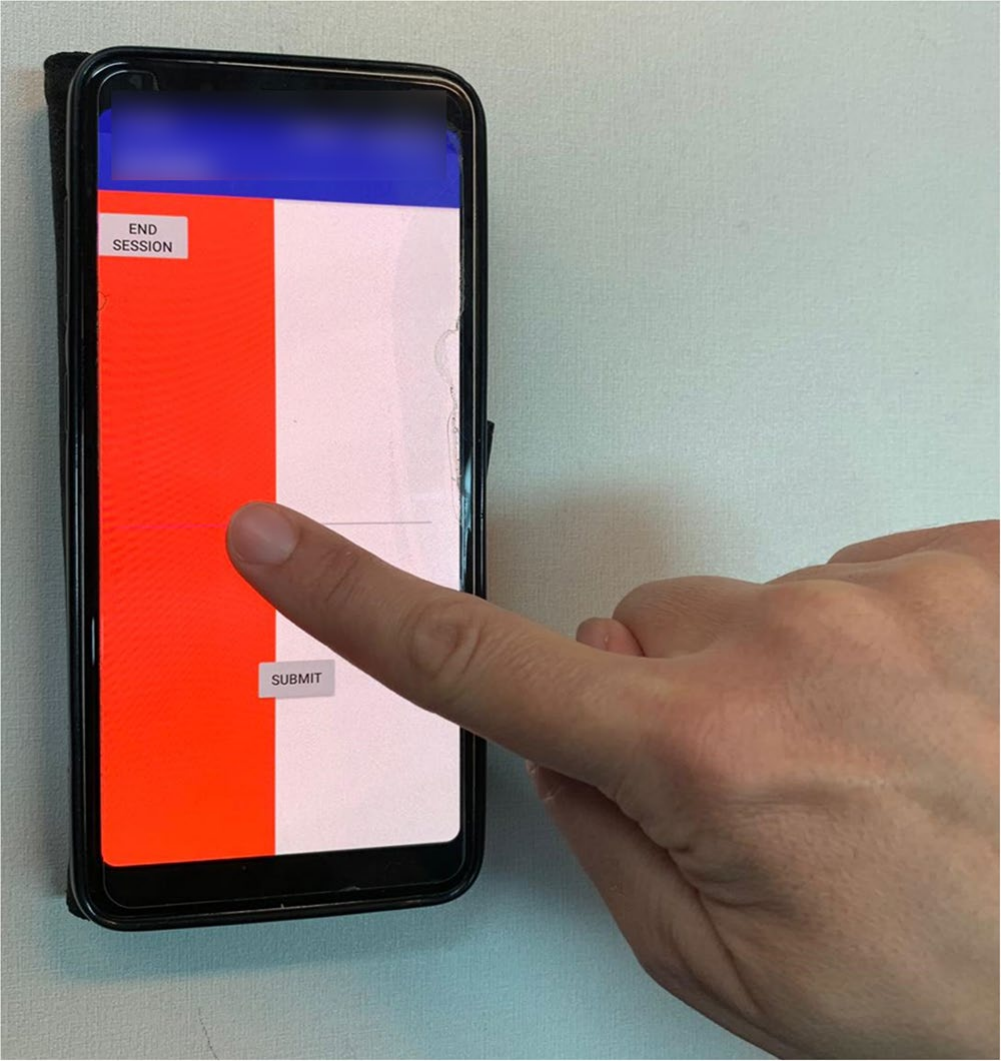
An illustration of the VAS application used to rate pain levels. In order to rate the intensity of pain, the participants were asked to slide their finger from left to right on the screen of the device. Sliding the finger revealed a red area that expanded as the participant slid his or her finger further to the right. The leftmost edge of the screen was defined to have a value of zero (no pain), and the rightmost edge was defined to have a value of 10 (the most intense pain).

The visual analog scale (VAS) is a form of direct scaling technique, in which line length is the response continuum^53^. The VAS has been reported as a valid and reliable measure for the intensity of pain^53^. Pain ratings were recorded by a custom-made application to digitally record the participants’ VAS responses, installed on a mobile device. Sliding the finger on the screen of the mobile device from left to right covers the corresponding portion of the screen in red (see Fig. 1), which, in turn, corresponds to the extent to which the participants experiences the stimulus as painful. The left end of the screen was defined as corresponding to ‘no pain sensation’, and the right end of the screen corresponded to ‘the most intense pain sensation imaginable’. The custom-made application translated the final horizontal finger location to a number on a scale from zero to 10.

### Measurements

#### Happiness state

Perceived happiness was evaluated using a VAS 10-cm line, printed on a sheet of paper, with 2 anchor points at its extremes, set as “not at all” (= 0) and “the most” (= 10), and participants were asked to mark on that scale, using a pen, how happy they felt. This method was found previously reliable and valid to measure the emotional state, including happiness^57^.

#### Empathic concern

Empathic concern was measured by the Empathic Concern subscale of the Interpersonal Reactivity Index (IRI). This is a 7-item questionnaire which assesses “other-oriented” feelings of sympathy and concern for others, found to have good reliability and sensitivity^58^.

#### Salivary oxytocin

Saliva samples of oxytocin were collected with salivates (Sarstedt, Rommels-dorft, Germany). Participants were asked to place a roll of cotton in their mouths, chew on it for a minute until it became saturated, and place it in a salivate tube. The samples were stored at −20 °C for approximately a week and then transported to the oxytocin laboratory where they were stored at −80 °C until they were assayed. Samples were thawed at room temperature for 10 minutes, followed by centrifugation (15 min., 3500 g, 4 $^\circ$C). Next, 1 ml of saliva was acidified with 1 ml of 0.1% trifluoroacetic acid (TFA) and centrifuged at 17000 ×g for 15 min at 4 °C. C18 Sep-Pak column (Waters, Ireland) were assembled onto vacuum manifold system (Waters, Ireland), and equilibrated with 1 ml of acetonitrile. Washing of columns was performed 5 times using TFA-H_2_O (in total: 15 ml), followed by applying of the supernatant onto the Sep-Pak vacuum manifold system, without vacuum, then an additional wash, as described. Elution of the samples was performed by applying 2 mL of an elution solution (95% acetonitrile 5% of 0.1% TFA-H2O) onto each column. Following extraction, collection and processing of saliva, measurements of human oxytocin concentrations were determined by an Enzyme-Linked Immunosorbent Essay (ELISA), using the Oxytocin ELISA kit (Abcam, Cambridge, UK). The ELISA plate was read at O.D. absorbance of 570 and 590 for Oxytocin (ELx808, Bio Tec Industries, VT). All samples were assayed and compared to a standard curve. Saliva concentration of the biomarkers was expressed as pg/ml.

#### Pain perception

Pain measurements were conducted at three time points during the experiments. The stimuli administered during these measurements were determined as follows:

#### Calibrating heat-pain intensity

To establish which temperatures elicit in each individual sensations of mild and strong pain, participants received a series of heat stimuli in a set of calibration trials. In each calibration trial, the starting temperature of the stimulator was 35 °C, and it increased at a rate of 2 °C/sec to a target temperature. The first target temperature was 40 °C. The target temperature was held for 6 sec, and participants were asked to rate the pain on the VAS. The temperature then returned to baseline (35 °C) by an active cooling mechanism. Following a 45-sec break, the subsequent trial was initiated. An interstimulus interval of 45 seconds was maintained and the contact probe was moved between stimulations to prevent sensitization. The target temperature was increased by 1 °C in each subsequent calibration trial until the participant reported a value of 6 (out of 10) on the VAS. The temperatures eliciting a value of 4 (mild pain) and a value of 6 (strong pain) on the VAS were documented and used for the rest of the experiment.

#### Pain measurements

In each of the three pain measurements, the temperatures eliciting a value of 4 and a value of 6 on the VAS were administered for 50 seconds with an inter-stimulus interval of 2 minutes. VAS pain ratings at the end of each stimulus was the outcome measure.

#### Structured interaction with PARO

During the 10 minutes of interaction with PARO, participants were asked to respond to questions, which encouraged them to examine PARO’s reactions (for example, indicate PARO’s reaction to petting it, to calling it by its name, etc.; See Supplementary Materials S1 for the full questionnaire). The goal of asking participants to fill out this questionnaire was to ensure that they spent the 10-minute session actively engaging with PARO.

#### Perceptions of the interaction with PARO

PARO’s perceived feelings, and participants’ feelings during the interaction with PARO, were evaluated using a 12-item custom-made questionnaire, to which participants responded using a 10-cm VAS line with 2 anchor points at its extremes, set to “not at all” (= 0) and “the most” (= 10) (see the full questionnaire in Supplementary Materials S2). The questionnaire was administered to the PARO group at the end of the experiment, at T4.

### Procedure

**Figure 2:**
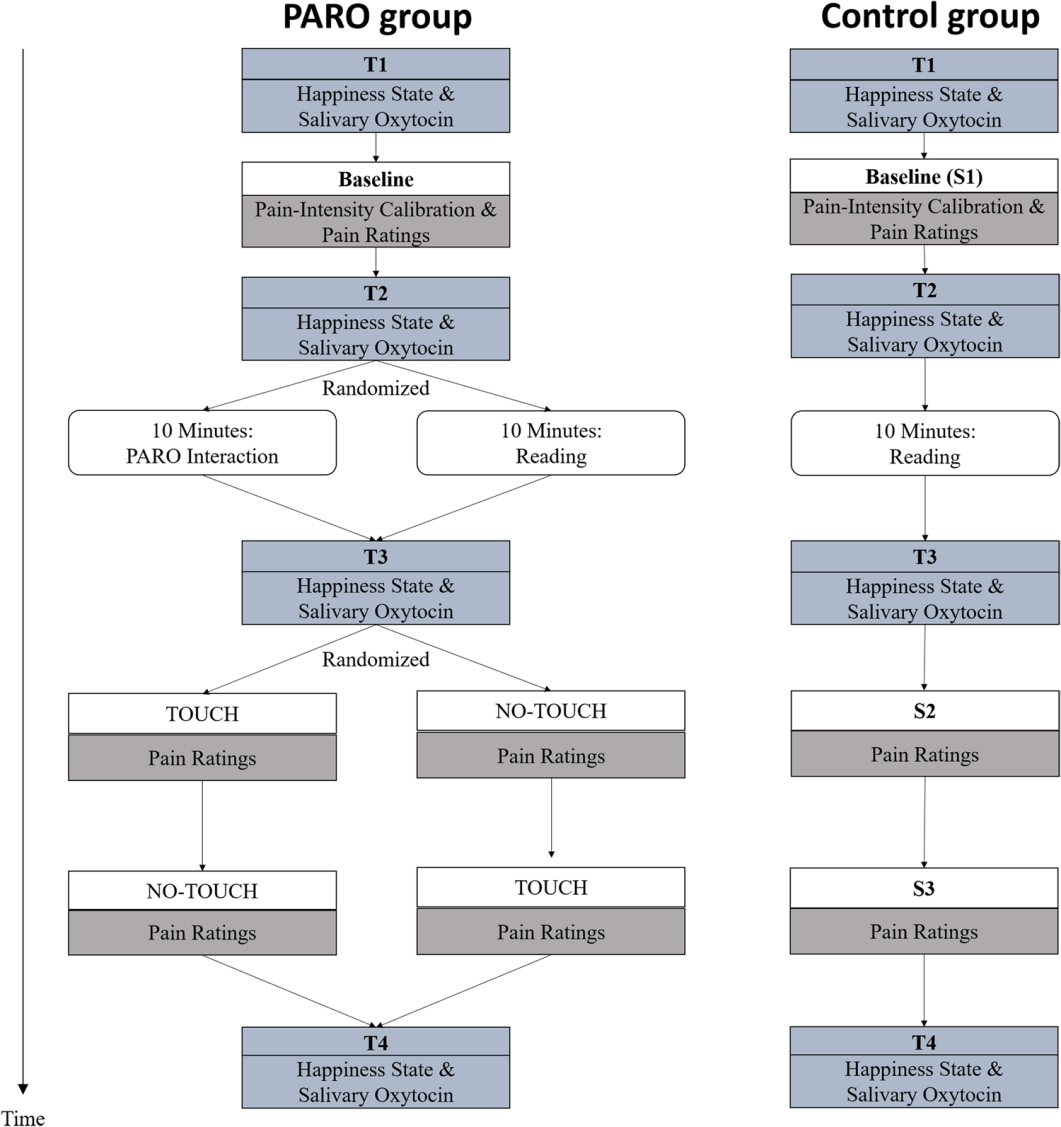
Flow chart of the experimental design.

**Figure 3:**
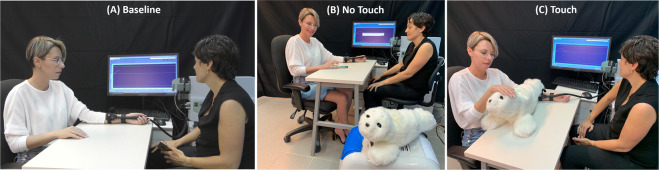
An illustration of the experimental setup. The participant (on the left) has the heat stimulator placed on her non-dominant arm, which is placed on the table. The experimenter (on the right) administers the accurate heat stimuli, and tracks them on the screen. (**A**) Baseline condition; PARO is not present. (**B**) No-Touch condition; PARO is present in the room, without physical contact with the participant. (**C**) Touch condition. PARO is placed on the table next to the participant, who touches it during the administration of the heat stimuli. In the control group, PARO was not present during the entire experimental session, as in (**A**).

Each participant was invited to a single testing session that lasted approximately 1 hour (see Fig. 2). The participants were instructed to avoid physical exercise and to refrain from smoking, eating or drinking (excluding water) for one hour before testing. Upon arrival, participants were divided semi-randomly to either the PARO or the control group. Testing took place in a quiet room. Temperature in the room was maintained at 25 °C. The participant sat in a comfortable armchair. Five minutes after arrival, the first happiness ratings and salivary oxytocin measurements were obtained (T1), followed by the pain-intensity calibration and the first pain measurement (Baseline; termed S1 in the control group, see Fig. 3A). Immediately after that, the second happiness ratings and salivary oxytocin measurements were obtained (T2). Participants in the PARO group then spent 10 min engaged in one of two activities they were semi-randomly assigned to: half of the participants in this group had a structured interaction with PARO (see below), and half were given an article to read on Maria Mitchel, an American astronomer. In the control group, all participants were given the article on Maria Mitchel to read during that 10-min period. Participants then underwent the third happiness ratings and salivary oxytocin measurements (T3). During T3, PARO was either present in the room (for the half of the PARO group which interacted with it for 10 minutes), or not present (for the half of the PARO group which read the article for 10 minutes). During the structured interaction with PARO, the experimenter introduced PARO to the participant and then left the participant alone in the room with PARO for 10 minutes. During the interaction, the participants completed a questionnaire that included questions about the interaction with PARO in order to ensure an active interaction experience (see section 3.5 above for details). The two subsequent pain measurements (Touch/No-Touch) in the PARO group were conducted while participants were either actively touching PARO (the ‘Touch’ condition, see Fig. 3C), or while PARO was co-present in the room with them, but with no physical touch between the participant and PARO (the ‘No-Touch’ condition, see Fig. 3B). The order at which the Touch and the No-Touch conditions were performed was semi-randomized across participants. The control group underwent the two subsequent measurements of pain intensity (S2 and S3) without ever encountering the PARO robot. Immediately after these, and while the participants were touching PARO, the forth happiness ratings and salivary oxytocin measurements were obtained (T4). Lastly, the participants completed the IRI questionnaire and rated their perceptions of the interaction with PARO.

The experimental protocol was approved by the Ethics Committee of the Ben-Gurion University of the Negev.

### Data analysis

Data were analyzed using IBM SPSS statistic software version 25 (IBM, Armonk, NY, USA). Continuous variables are described as means ± SD. Sample size was calculated using G-Power^59^. For a sample size of 83 individuals, if α  =  0.05 statistical power is 89%. All data underwent Kolmogorov-Smirnov analysis for normality of distribution. Parametric and nonparametric analyses of variance with corrected post hoc tests were used to evaluate the effect of experimental phase (T1/T2/T3/T4) and of group (PARO/Control group) on perceived happiness and on oxytocin levels and the effect of condition (Baseline/Touch/No-Touch in the PARO group and S1/S2/S3 in the control group) on pain ratings. Correlations between pairs of variables were calculated with Pearson’s r; p < 0.05 was considered significant. The Bonferroni correction was applied to multiple comparisons, where needed.

## Results

### Participants’ perceptions of the interaction with PARO

#### PARO’s perceived feelings during the interaction

**Figure 4:**
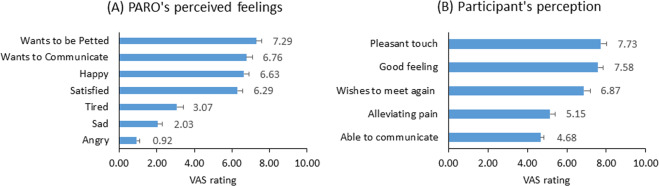
Participants’ perceptions of the interaction with PARO. (**A**) PARO’s perceived feelings, as evaluated by participants in the PARO group at the end of the experiment (at T4). (**B**) Participants’ impressions from their interaction with PARO (PARO group), which they reported at the end of the experiment (at T4). Values denote mean of visual analogue scale (VAS; 0–10) ± SEM.

The participants perceived PARO’s feelings during the interaction as happy (6.6 ± 2.2), satisfied (6.3 ± 2.2), wants to be petted (7.3 ± 2.2) and wants to communicate (6.8 ± 2.6). Low ratings were given to PARO feeling tired (3.1 ± 2.7), sad (2.0 ± 1.9) and angry (0.9 ± 1.1) (Fig. 4A). These perceptions of PARO’s feelings were recorded once, at T4.

#### Participants’ feelings during the interaction with PARO

The participants gave high ratings to feeling good in the presence of PARO (7.6 ± 1.8), to pleasant sensation while touching PARO (7.7 ± 2.0) and to their willingness to meet PARO again (6.9 ± 2.7). Intermediate ratings were given to the question if PARO helped to reduce pain (5.2 ± 2.7) and to the question if they were able to communicate with PARO (4.7 ± 3.0) (Fig. 4B). There were significant correlations between the participants’ empathic concern and: (1) their good feelings in the presence of PARO (r = 0.27, p = 0.021) and (2) their pleasant sensation while touching PARO (r = 0.30, p = 0.012).

### The effect of the interaction with PARO on the participants’ emotional state

**Figure 5:**
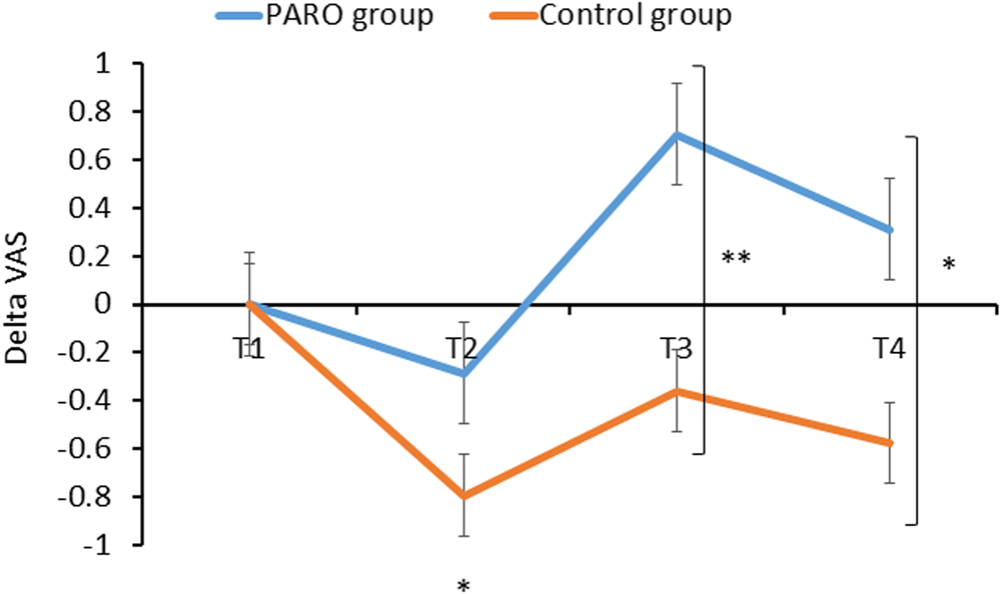
The change in perceived happiness at the four epochs of the study in the PARO and the control groups. A significant difference between groups was found at T3 (**p < 0.01) and T4 (*p < 0.05). At T2 happiness decreased significantly in the control group compared to T1 (p < 0.05). In the PARO group the change in happiness at T2 (compared to T1) was not significant, p = 0.06. At T3 Happiness increased significantly compared to T1 only in the PARO group (****p < 0.0001). Values denote mean of Δ visual analogue scale (VAS; 0–10) ± SEM. **T1** – upon arrival; **T2** – following the first pain-perception measurements; **T3** – following 10 mins of either reading or interacting with PARO; **T4** – completed while touching PARO (in the PARO group only), following the third, and last, pain-perception measurements.

A significant main effect of the experimental phase (T1/T2/T3/T4) was found for happiness ratings [F(3,83)=4.84, p < 0.05(. The effect of group (PARO/Control) was not significant [F(1,83)=1.71, p = 0.19]. However, the interaction phase*group was significant [F(3,83)=3.73, p < 0.05]. Post hoc comparisons revealed that there were similar ratings between groups at T1 (5.6 ± 1.9 in PARO and 5.6 ± 1.7 in controls, t(81)=0.62, p = 0.47) and T2 (5.3 ± 2.2 in PARO and 4.8 ± 2.3 in controls, t(81)=0.93, p = 0.18). However, at T3 there was an increase in happiness, compared to T1, in the PARO group (6.3 ± 1.9, t(62)= 3.52; t(19)=0.87, p < 0.001) but not in the control group (5.2 ± 2.3, t(19)=1.28, p = 0.20). As noted above, the PARO group included both those who spent the 10 minutes interacting with PARO, and those who read the article during the 10-min period. The difference between groups at T3 was significant (t(81)=2.08, p < 0.05). At T4 the happiness ratings remained higher than T1 in the PARO group (5.9 ± 2.2, (t(62)=1.44, p < 0.001) and did not change significantly in the control group (5.0 ± 2.4, t(19)=1.28, p = 0.11). The difference between groups at T4 did not reach significance (p = 0.05, Fig. 5). There were significant correlations between PARO’s perceived feelings and the change in happiness from T1 to T4 (see Table 1). The more participants perceived PARO to have more positive feelings, the happier they reported feeling themselves.

**Table 1:** Correlations between participants’ perceptions of PARO’s feelings, and the change in their happiness in the Touch condition compared to Baseline.

	PARO’s perceived feelings		
	Satisfied	Happy	Sad
Δ Happiness (0–10)	0.38** p < 0.005	0.37** p < 0.005	−0.57** p < 0.005

Shown here are the r values. **p < 0.005.

### The effect of the interaction with PARO on salivary oxytocin levels

**Figure 6:**
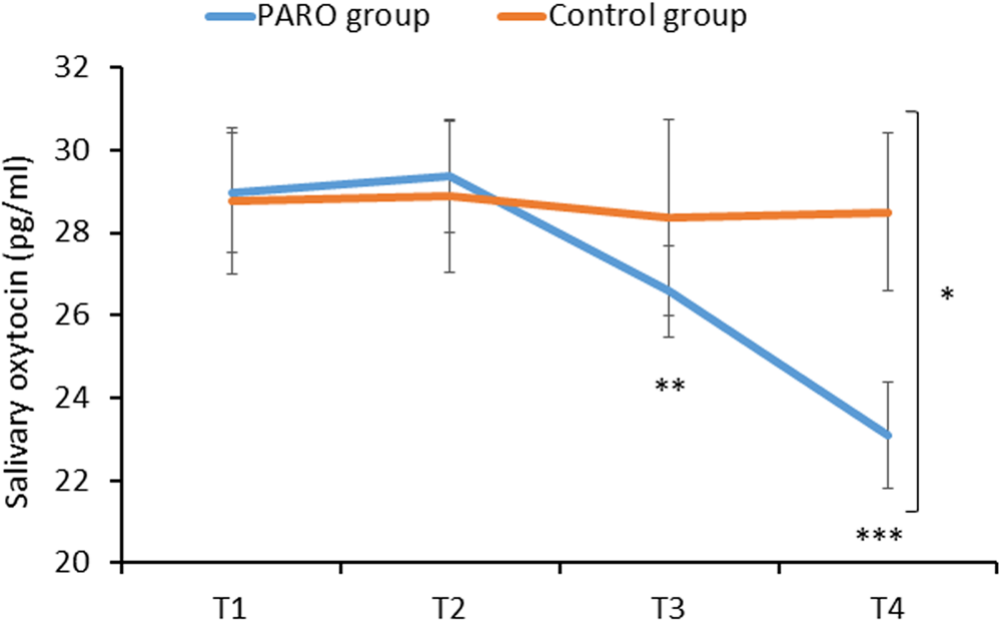
The change in oxytocin levels at the 4 epochs of the study in the PARO and the control groups. Oxytocin levels decreased significantly from T1 to T3 (**p < 0.01) and further from T1 to T4 (***p < 0.001) only in the PARO group. The difference in oxytocin levels between the groups was significant at T4 (*p < 0.05). Values denote mean salivary oxytocin levels (pg/ml) ± SEM. **T1** – upon arrival; **T2** – following the first pain-perception measurements; **T3** – following 10 mins of either reading or interacting with PARO; **T4** – completed while touching PARO (in the PARO group only), following the third, and last, pain-perception measurements.

A significant main effect of the experimental phase (T1/T2/T3/T4) was found for oxytocin levels [F(3,82)=6.54, p < 0.01]. The effect of group (PARO/Control) was not significant [F(1, 82)= 0.49, p = 0.49]. However the interaction phase*group was significant [F(3,82)=5.16, p < 0.01]. Post hoc comparisons revealed that the levels of oxytocin were similar at time T1 in the PARO group (29.0 pg/ml±11.4) and in the control group (28.8 pg/ml ±8.0, t(81)=0.72, p = 0.47). In both groups oxytocin levels did not change at T2. However, in the PARO group, oxytocin levels decreased significantly at T3 to 26.6 pg/ml ±8.8 (t(61)=2.57, p < 0.01, a decrease of 2.8 pg/ml ±8.3) and decreased significantly further at T4 compared to T1 to 23.1 pg/ml ±10.2 (t(62)=5.73, p < 0.0001, a decrease of 5.9 pg/ml ±8.1). However, in the control group oxytocin levels did not change significantly at T3 (28.4 pg/ml ±10.5, t(19)=0.29, p = 0.39) or at T4 (28.5 pg/ml, t(19)=0.26; p = 0.40). The difference between groups in the reduction of oxytocin levels was significant at T3 (t(81)=0.75, p < 0.001) and T4 (t(81)=2.14, p < 0.05, see Fig. 6). As noted above, the PARO group included both those who spent the 10 minutes interacting with PARO, and those who read the article during the 10-min period.

There was a significant negative correlation between oxytocin levels at T4 and the participants’ willingness to meet PARO again (r = −0.46, p < 0.05).

### The effect of the interaction with PARO on pain perception

#### Mild pain

**Figure 7:**
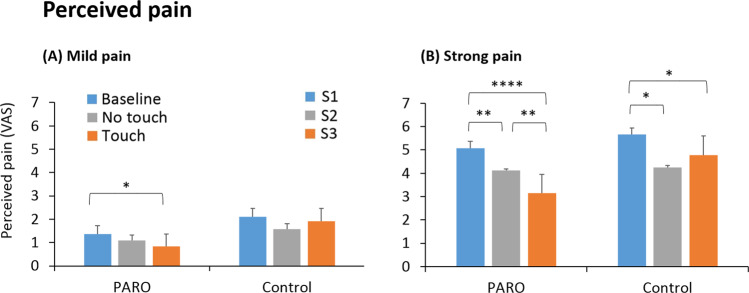
The change in pain ratings across the three pain measurements in the PARO and the control groups. (**A**) Mild pain: In the PARO group, pain ratings decreased in the Touch condition compared to Baseline (*p < 0.05). (**B**) Strong pain: In both the PARO and the control groups, pain ratings decreased in the No-Touch/S2 and Touch/S3 conditions compared to Baseline/S1. In the PARO group there was a greater decrease in the Touch compared to the No-Touch condition (**p < 0.01). Values denote mean visual-analogue scale (VAS) ratings (0–10) ± SEM.

A significant effect of condition (Baseline/Touch/No-Touch) was found in the PARO group (F_2,62_ = 4.33, p < 0.05) while no effect of condition (S1/S2/S3) was found in the control group (F_2,20_ = 1.16, p = 0.33). Post hoc tests revealed that in the PARO group there was a significant decrease in pain ratings from Baseline (1.4 ± 1.6) to the Touch condition (0.8 ± 1.4, t(62)=2.59, p < 0.05). No significant difference from Baseline was found at the No-Touch condition (1.1 ± 1.6, t(61)=1.74, p = 0.87, Fig. 7A). In other words, participants rated their pain sensation as significantly lower when touching the PARO robot, compared to Baseline pain ratings. When the robot was only co-present in the room with them, and there was no physical contact with it, their pain ratings were not significantly different from Baseline.

The decrease in mild-pain ratings from Baseline to Touch condition in the PARO group was significantly correlated with the perceived pain-alleviating effect of PARO (r = −0.34, p < 0.005; see Table 2) and with the level of salivary oxytocin at T4 (r = 0.24, p < 0.05).

**Table 2:** Correlations between participants’ perceptions of the interaction with PARO, and the change in pain ratings in the Touch condition compared to Baseline.

	Participant’s perceptions of the interaction with PARO				
	Good feelings	Pleasant touch	Able to communicate	Alleviating pain	Wish to meet again
Δ Mild pain	NS	NS	NS	−0.34** p < 0.005	NS
Δ Strong pain	−0.31* p < 0.01	NS	NS	−0.33** p < 0.005	−0.37** p < 0.005

Shown here are the r values. *p < 0.01, **p < 0.005, NS = not significant.

#### Strong pain

A significant effect of condition (Baseline/Touch/No-Touch) was found in the PARO group (F_2,62_ = 17.87, p < 0.0001). The effect of condition (S1/S2/S3) was also significant in the control group (F_2,20_ = 7.78, p < 0.01). Post hoc tests revealed that in the PARO group there was a decrease in pain ratings from Baseline (5.1 ± 2.4) both to the No-Touch condition (4.1 ± 2.7, t(62)=3.44, p < 0.01) and to the Touch condition (3.1 ± 2.5, t(61)=6.23, p < 0.0001). The decrease in pain ratings was significantly greater in the Touch condition compared to No-Touch condition (t(61)=2.56, p < 0.01). In the control group, there was also a decrease in pain ratings from S1 (5.7 ± 2.7) to S2 (4.3 ± 3.2, t(19)=3.59, p < 0.05) and to S3 (4.8 ± 3.0, t(19)=2.64, p < 0.05). However, no significant difference was found between S2 and S3 (t(19)=1.51, p = 0.49, Fig. 7B).

The extent of the decrease in strong-pain ratings from Baseline to the Touch condition in the PARO group was significantly correlated with the participants’ perceived pain-alleviating effect of PARO (r = −0.33, p < 0.005), their positive feelings with respect to PARO (r = −0.31, p < 0.01) and the wish to meet PARO again (r = −0.37, p < 0.005; see Table 2).

#### High and low communication with PARO

In order to further investigate the effect of the interaction with PARO on emotions and pain perception, we divided the participants in the PARO group into high communicators (HC) and low communicators (LC). The division into the two groups was made using the median value of the perceived ability to communicate with PARO (4.7).

**Figure 8:**
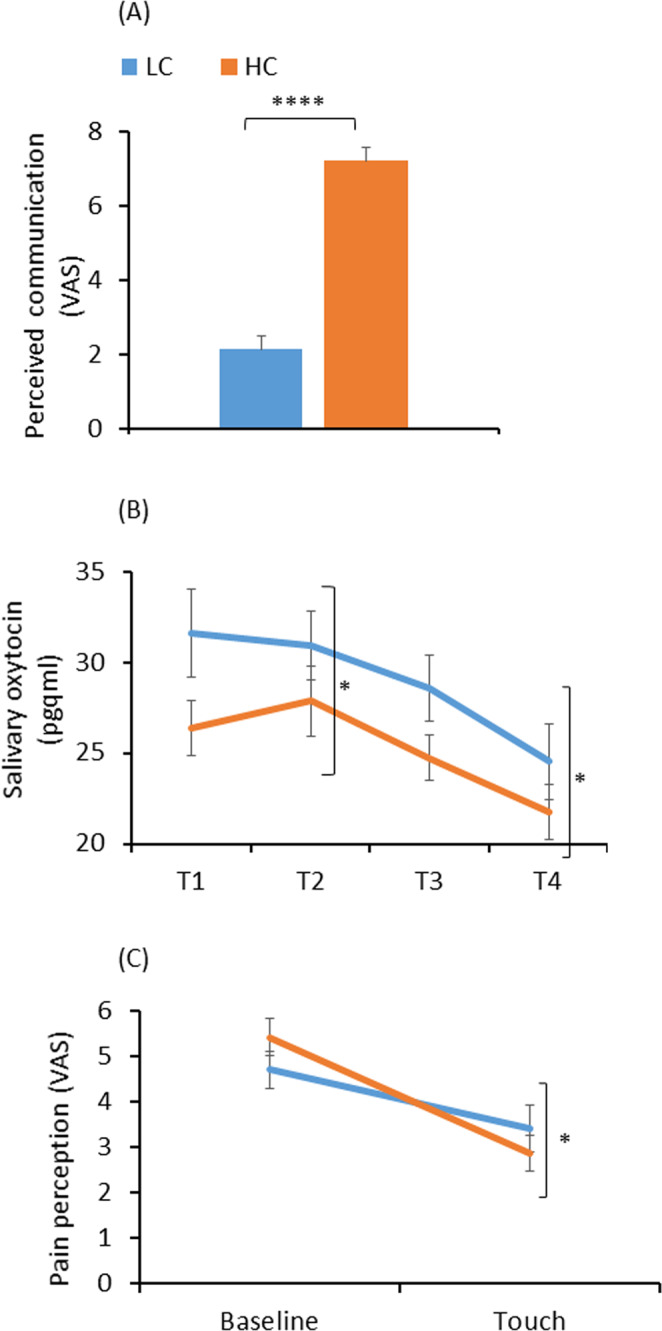
Differences between low communicators (LC) and high communicators (HC). (**A**) perceived ability to communicate with PARO (****p < 0.0001); (**B**) oxytocin levels. Significant difference at T1 and T3 (*p < 0.05); (**C**) Strong-pain ratings. The decrease in pain ratings from Baseline to Touch condition was greater in HC compared to LC (*p < 0.05). Values denote mean visual analogue scale (VAS; 0–10) (**A,C**) and mean salivary oxytocin (pg/ml) (**B**) ±SEM. **T1** – upon arrival; **T2** – following the first pain-perception measurements; **T3** – following 10 mins of either reading or interacting with PARO; **T4** – completed while touching PARO (in the PARO group only), following the third, and last, pain-perception measurements.

The mean communication ratings of HC (n = 31) and LC (n = 32) was 7.2 ± 1.9 vs. 2.1 ± 1.9 respectively (p < 0.0001; Fig. 8A). There was no significant difference between the subgroups in happiness ratings. However, oxytocin levels were lower in HC compared to LC. The difference between subgroups was significant at T1 (31.6 ± 13.4 pg/ml in LC and 26.4 ± 8.6 pg/ml in HC;t(61)=1.83, p < 0.05) and at T3 (28.6 ± 10.2 pg/ml in LC and 24.7 ± 7.0 pg/ml in HC;t(61)=1.75, p < 0.05; Fig. 8B). There was no difference between the HC and the LC in mild-pain ratings. However, the decrease in strong-pain ratings from Baseline to Touch condition was significantly greater in HC (2.5 ± 2.7) compared to LC (1.3 ± 1.8, t(61)=0.85, p < 0.05; Fig. 6C).

## Discussion

The results revealed that interacting with the baby-seal PARO robot induced an increase in perceived happiness, a decrease in oxytocin levels and a reduction in pain ratings to both mild and strong heat stimuli. Moreover, the reduction in pain ratings was greater when *touching* the robot in contrast to being in the mere presence of it. The reduction in pain ratings was correlated with the participants’ positive perceptions of the interaction with PARO, and with oxytocin levels. This is the first study, to the best of our knowledge, to demonstrate a *decrease* in salivary oxytocin during social interaction.

### The effect of the interaction with PARO on emotions

Happiness ratings increased significantly after the interaction with PARO. Happiness is considered to be a central human goal across cultures^60^ and is an important determinant of well-being^61^. As humans are inherently social, happiness is often related to social interaction. An extensive body of research emphasizes the key role of positive social connections in humans’ perceived happiness, satisfaction and stress buffering e.g^62–65^.. It appears that the effect of social connections on happiness is not exclusive to human-human interactions. Positive emotions, including happiness, were also found to be associated with interaction with companion animals^66–68^ and, in the recent two decades, with interactions with social robots^69,70^. Although there is broad evidence on the link between human-animal and human-robot social interactions and perceived happiness, stress and well-being, the majority of the studies examined either children, the elderly or hospitalized populations while only few focused on healthy adult population^71^; for review see^67,72^. Moreover, we could find only two controlled studies examining, as we did, the effect of controlled social interaction on the emotional state. Both of these investigated the effect of interaction with a social entity on the emotional state of children, using a pet dog^73^ or the PARO robot^74^. Both studies showed that the interaction with the social entity (pet dog or PARO robot) increased positive emotions, including happiness. Our current study, adds to the existing body of knowledge, in demonstrating that interaction with the PARO robot is effective in increasing perceived happiness also in healthy adults. The correlation we found between participants’ positive perceptions of PARO’s feelings and the increase in happiness further support this finding.

### The effect of the interaction with PARO on oxytocin levels

In this study, oxytocin levels decreased only in the PARO group while no change in oxytocin levels was found in the control group throughout the experiment. In the PARO group, there was an inverse correlation between oxytocin levels and the sense of connection with PARO: the lower oxytocin levels were at T4, the higher was the participant’s willingness to meet it again. This is the first study to examine endogenous oxytocin levels during human-robot interaction (HRI). Over the last decade, several studies have examined the role of oxytocin in human relations. The strongest relationship was found with stress, with several studies showing that an increase in physiological and psychological stress is associated with an increase in endogenous oxytocin, whether in saliva or plasma^75–79^. Similarly, removal of a stressor induces a rapid decrease in oxytocin^75–77^. Moreover, a recent meta-analysis concluded that even a novel laboratory context may induce a significant oxytocin increase^80^. The results of these studies suggest that participants arriving to the novel laboratory setting in the current study may have experienced an increase in oxytocin levels during the first measurement of oxytocin (T1). The reduction in oxytocin levels at T3 and T4 in the PARO group may have resulted from participants feeling more at ease due to the interaction with PARO. That is, it appears that the interaction with PARO led to a decrease in stress and an accompanying rapid decrease in oxytocin levels. In contrast, control participants appear to have remained at higher levels of alertness throughout the experimental session, as evidenced by their unchanged salivary oxytocin levels. These findings support previous findings on the role of oxytocin as an important hormone in the stress system, which shows a positive association with cortisol^80^, known to respond to social stimuli.

Another vein of research points to a positive association between oxytocin and social interaction, which at first seems to be at odds with the current findings. These studies focus on positive interactions with romantic partners or with family members, such as during parent-infant bonding^25,81^ and romantic relationships^20,31,82^ However, interactions with non-close others seem to be less effective in activating oxytocin release. For example, a study conducted with chimpanzees found that oxytocin elevation was specific to grooming kin or potential mating partners while no increase in oxytocin was found for grooming chimpanzees that did not have a strong social bond^83^. Among humans, Feldman *et al*.^25^ demonstrated increase in both salivary and plasma oxytocin only among mothers displaying high levels of affectionate contact during mother-infant interaction. Thus, it appears that there is a U-shaped relationship between oxytocin secretion, stress, and social bonding. The interaction with PARO appears to have reduced the stress level of participants, leading to a reduction in salivary oxytocin levels, compared to controls, who did not meet PARO, and their salivary oxytocin levels remained constant. Indeed, several studies show that the effect of oxytocin on behavior is context-dependent and may induce, at the same time, bonding and trust toward in-group members, while increasing aggression and mistrust toward out-group members;^84–92^ for a review see^93^. For example, administration of nasal oxytocin enhanced cautious behavior and feeling of mistrust during a social dilemma^88^ as well as promoted aggressive behavior during a social game^89^.

These results suggest that a decrease in oxytocin levels may facilitate trust and sociability with members of an out-group. Since individuals may identify robots as out-group members^94^, the observed decrease in oxytocin levels might be related to the participants’ inclination to lower their aggression toward it and to establish their trust in it. The negative correlation found between oxytocin levels and participants’ willingness to meet PARO again, further support this explanation.

In the current study, we had nearly equal numbers of males and females in both the PARO and the control groups, and we did not test the effects of gender on oxytocin levels. The observed effect of the interaction with PARO on oxytocin levels thus appears to be *in addition* to any gender effects, if those exist. As endogenous oxytocin levels appear to depend on a variety of factors^95^, it would be instructive to test, in a future study, whether gender plays a role in endogenous oxytocin levels when interacting with a social robot.

### The effect of the interaction with PARO on pain perception

The results reveal diminished levels of pain during the interaction with PARO compared to baseline and compared to the control group. The decrease in pain ratings was more pronounced in the Touch condition compared to the No-Touch condition. Thus, this study highlights remarkable benefits of human-robot social interactions on pain perception. In accordance with our findings, previous data indicate that interaction with PARO or humanoid robot reduces clinical pain among pediatric patients^96^, cancer patients^49^ and children undergoing medical procedures^50,97^. It is important to note that this is the first study to examine the effect of HRI on pain perception among healthy adults. Moreover, it is the first to examine the effect of HRI on pain in a controlled laboratory setting. There are several possible explanations to our findings. First, our finding that touching PARO had the strongest effect in alleviating pain compared to its presence in the room without any physical contact and compared to the control condition, where participants did not meet PARO at all, highlights the effect of social touch on pain alleviation. This is the first study to examine the effect of touching a robot on experimentally induced pain perception. However, among humans, previous studies have found that holding a partner’s hand decreased pain ratings compared to the mere presence of the partner in the room, a stranger’s touch or no interaction, and compared to squeezing a ball^33,34^. In the current study, the participants gave high ratings to their positive feelings towards PARO. Research indeed suggests that social HRI, and particularly touching a robot, induce positive feelings towards it^98,99^. Thus, we speculate that touching PARO enabled participants to form an emotional connection with it, which led to similar beneficial outcomes on pain perception as was found during a partner’s touch^33,34^. It can be also speculated that the interaction with PARO attenuated pain by promoting relaxation. Indeed, recent evidence suggest that touching a robot can reduce stress^99^. Moreover, Robinson *et al*.^100^ showed that stroking PARO reduced blood pressure and heart rate and was accompanied by feelings of happiness and relaxation. Furthermore, there is evidence that high psychosocial stress enhances pain^101,102^. Indeed, some relaxation techniques were found effective in attenuating pain^103–105^. Taken together, it is possible that the interaction with PARO led to a more relaxed state of mind and thus reduced pain perception.

Another possible explanation of our finding is that the interaction with PARO distracted the participants away from pain. Changing the focus of attention away from painful stimuli was shown to be efficacious in altering pain perception^106–108^. Thus, it is possible that having a novel stimulus like PARO in the room distracted the participants away from pain, leading to reduced pain ratings. However, the presence of PARO in the room without any physical contact did not affect mild-pain ratings and affected strong-pain ratings to a lesser extent than did the condition when participants touched PARO. Notably, PARO was active in the No-Touch condition: participants looked at it and were aware of the sounds and movements it made. Thus, if distraction is at play here, then touching PARO provides a more effective distraction than its mere presence in the room. Furthermore, it is likely that there is more at play here than mere distraction, as evidenced by the significantly more pronounced effect that the interaction with PARO had on pain perception in the high-communicators group, suggesting the social aspect of the interaction played a role in modulating pain perception.

One may also speculate that the effect of touching PARO’s fur on pain perception stems from the tactile stimulation of touching a soft object. It was previously demonstrated that tactile stimulation could decrease nociceptive input^109,110^. This effect is attributed to the capability of sensory fibers to suppress the transmission of nociceptive input^111,112^. However, this analgesic effect strongly depends on the relative spatial location of the tactile and nociceptive stimuli within the same dermatome. In general, the closer the nociceptive and tactile stimuli, the more powerful the analgesia^109,110^. In this current study, participants touched PARO in a remote area to the nociceptive stimuli (the arm they used for petting PARO was the opposite arm from the one on which the heat stimuli were applied), thus, this explanation is less probable.

Willemse & Erp^99^ found that touching a robot is effective in increasing the perceived intimacy with the robot and reducing physiological stress response, and is not dependent on whether there is a prior session of interaction with the robot. Thus, it is likely that, in our experiment, the social touch with PARO induced an effect on both emotions and pain perception regardless of the presence of a preliminary bonding session. This is clinically important since using a robot in the clinical field to improve positive emotions and reduce pain does not seem to require prior acquaintance and hence is easier to implement. Moreover, the effect of touching PARO on pain reduction was demonstrated both in mild and strong pain intensity. This finding further illustrates the clinical potential of human-robot social touch on pain management.

The control group also experienced a reduction in pain ratings compared to baseline levels after reading the Wikipedia article. However, this reduction was significantly smaller compared to the reduction in pain perception in the PARO group. This reduction can be explained by a regression to the mean^113^, or that reading the article induced a certain level of relaxation and hence reduced pain ratings somewhat.

We further found positive correlations between the empathic concern scores and the participants’ positive perceptions of the interaction with PARO. It has been shown that activation of brain networks involved in the perception of empathy are associated with both pain and social touch^24,114^. This suggests an interesting basis for future exploration of the connection between empathic concern level, and the pain-alleviating effect of touch in human-robot social interactions. It is possible that high empathic ability enables participants to embrace positive social relationship with PARO, which would amplify the pain-alleviating effect of touching it. This research direction would dovetail with a recent study showing that the empathic abilities of the partner predict the magnitude of pain reduction during touch between partners^33^.

Further exploring interpersonal traits, the division of the PARO group according to their perceived ability to communicate with PARO revealed that participants classified as “high communicators” exhibited greater pain reduction as well as lower oxytocin levels compared to “low communicators”. These results, along with the significant correlations between participants’ perceptions of the interaction with PARO and the change in pain ratings, demonstrate that the effect of touching PARO on pain perception largely depends on the participant’s ability to form a social connection with PARO. It was found that the ability to communicate contributes to the extraversion personality trait^115^. It was further shown that a short human-robot social interaction can predict extraversion in a way comparable to the predictive power of human-human interactions^116^. Other studies demonstrated that high extraverted people exhibit higher affective trust^117^ and obtain greater benefits from social connection^64,117^, particularly from social touch^117^. Our findings thus add to the current literature in demonstrating that high communicators reap greater benefits from the interaction with PARO.

In summary, this study indicates that social touch with PARO robot alleviates pain, increases happiness state and decreases oxytocin levels. Participants with higher perceived ability to communicate with PARO display greater pain alleviation as well as lower oxytocin levels. These findings reveal a profound effect of human-robot social interaction on pain and emotions and hence extend the current knowledge on the impact of social touch on pain and emotions, and offer new strategies for pain management and for improving well-being.

## Supplementary information

Supplementary Materials.

## Data Availability

The datasets analyzed during the current study are available from the corresponding author on reasonable request.
