# Supplementary material for: Touching the social robot PARO reduces pain perception and salivary oxytocin levels
Source: Sci Rep. 2020 Jun 17;10:9814. doi: 10.1038/s41598-020-66982-y (PMC7299999; doi:10.1038/s41598-020-66982-y)
Supplement: Supplementary file 1 — Supplementary Materials. [file 41598_2020_66982_MOESM1_ESM.pdf]

**Touching the social robot PARO reduces pain perception and salivary oxytocin levels**

Nirit Geva, Florina Uzefovsky, Shelly Levy-Tzedek*

# Supplementary Materials

**Questionnaire 1 – Getting to know PARO**

***Please indicate PARO's reaction to the following:***

When I pet its back several times ______________________________

When I pet its face several times ______________________________

When I pet its front flippers several times ______________________________

When I pet its back flippers several times ______________________________

When I call it by its name several times ______________________________

When I call it by its name when it cannot see me ______________________________

When I touch its whiskers (gently – no pulling) ______________________________

***Please mark with a circle whether you observed PARO perform any of the following:***

Lift its gaze upwards ··· Yes / No

Place its head in your lap ··· Yes / No

Move its front flippers ··· Yes / No

Move its back flippers ··· Yes / No

Open and close its eyes ··· Yes / No

Make sounds ··· Yes / No

**Questionnaire S1. Getting to know PARO.** This questionnaire was given to half of the participants in the PARO group – those who had a 10-min interaction with it. They were asked to fill it out during the 10-min interaction.

**Questionnaire 2**

| ***How does PARO feel?*** | **Not at all** | **The highest degree** |
|---|---|---|
| Happy | | |
| Satisfied | | |
| Sad | | |
| Angry | | |
| Tired | | |
| Wants to be petted | | |
| Wants to communicate | | |
| ***How did you feel during the interaction?*** | | |
| Did you feel good with PARO? | | |
| Is it pleasant to touch PARO? | | |
| Did you feel you could communicate with PARO? | | |
| Did PARO help to reduce pain? | | |
| Would you like to meet PARO again? | | |

**Questionnaire S2. Perceptions of the interaction with PARO**. Participants were asked to mark on a 10-cm VAS line (with no numbers on it) how they perceived PARO's and their own feelings. The ends of the VAS line were marked as “not at all” (evaluated offline as = 0) and “the most” (evaluated offline as = 10).
